# Supplementary material for: Adaptive Support Ventilation Attenuates Ventilator Induced Lung Injury: Human and Animal Study
Source: Int J Mol Sci. 2019 Nov 21;20(23):5848. doi: 10.3390/ijms20235848 (PMC6929029; doi:10.3390/ijms20235848)
Supplement: Supplementary file 1 [file ijms-20-05848-s001.pdf]

SUPPLEMENTARY FILE

Adaptive support ventilation attenuates ventilator induced lung injury: Human and Animal study

Yu-Ling Dai, MSc, RRT, Chin-Pyng Wu, MD, PhD, Gee-Gwo Yang, MD, PhD, Hung Chang, MD, PhD, Chung-Kan Peng, MD, PhD, Kun-Lun Huang, MD, PhD,

Materials and Methods

Human studies

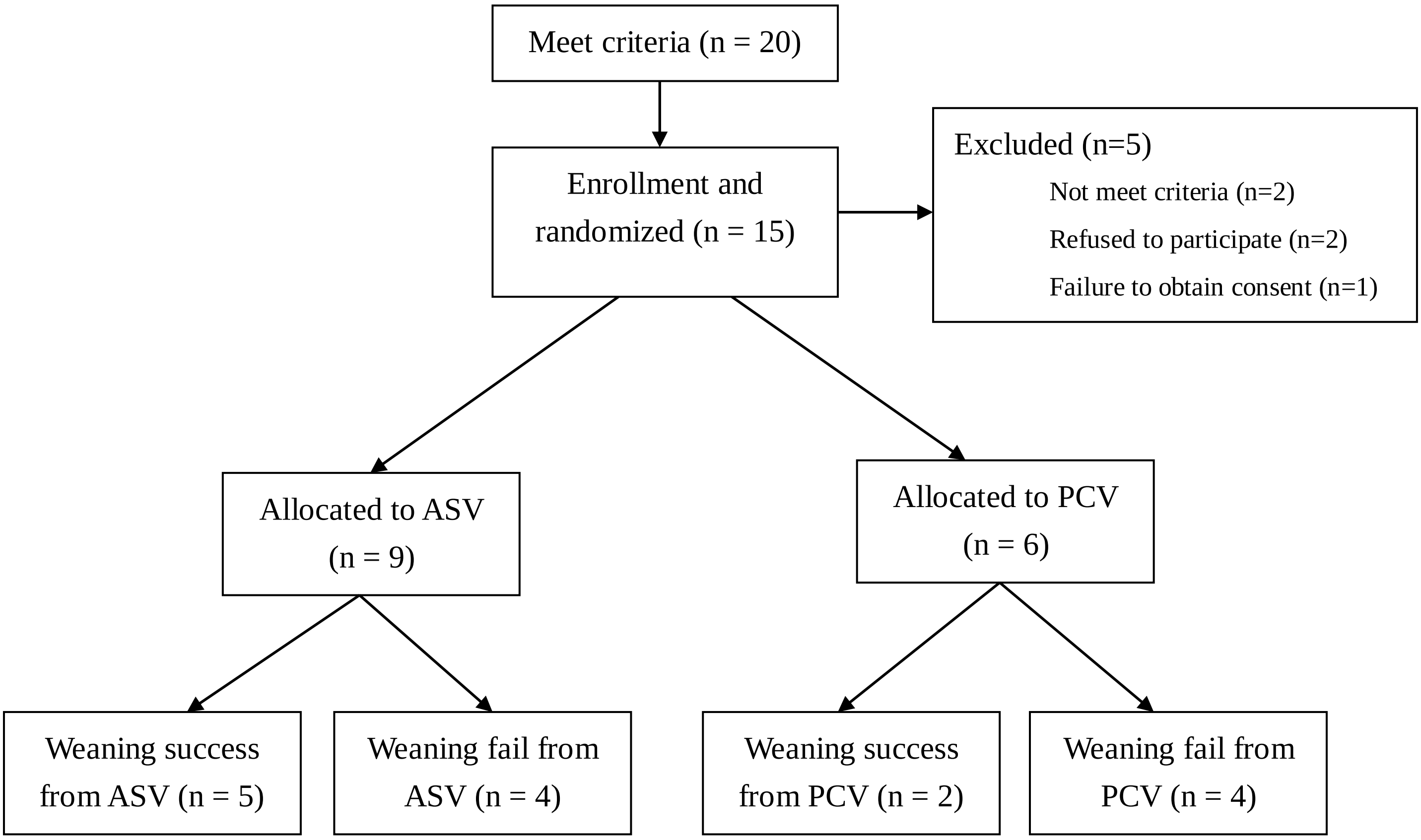

Figure S1. Flow chart for the patient recruitment in the ASV group and the PCV group. ASV = adaptive support ventilation; PCV = Pressure control ventilation

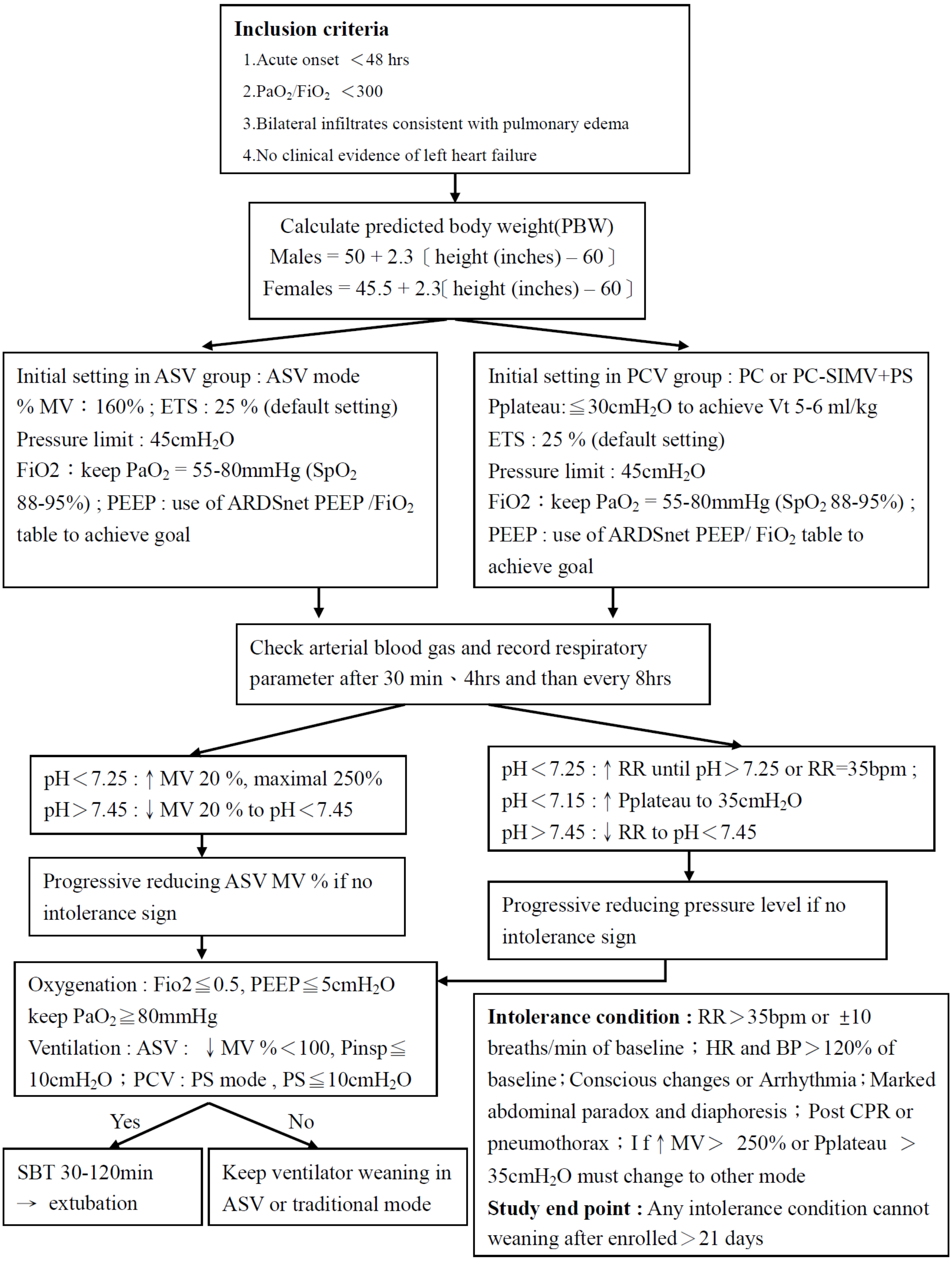

Figure S2. Ventilation setting protocol for adaptive-support ventilation (ASV) and pressure control ventilation (PCV).

PC =pressure control; PC-SIMV+PS= pressure control-synchronized intermittent mandatory ventilation and pressure support; PaO_2 = arterial oxygen tension; FiO_2 = fraction of inspired oxygen; ETS = expiratory trigger sensitivity; MV = minute ventilation; Pplateau = inspiratory plateau pressure; Pinsp = inspiratory pressure; PEEP = positive end-expiratory pressure; RR = respiratory rate; PS = pressure support; SBT = spontaneous breathing trial; HR = heart rate; BP = blood pressure; CPR = cardiopulmonary resuscitation.

Animal experiments

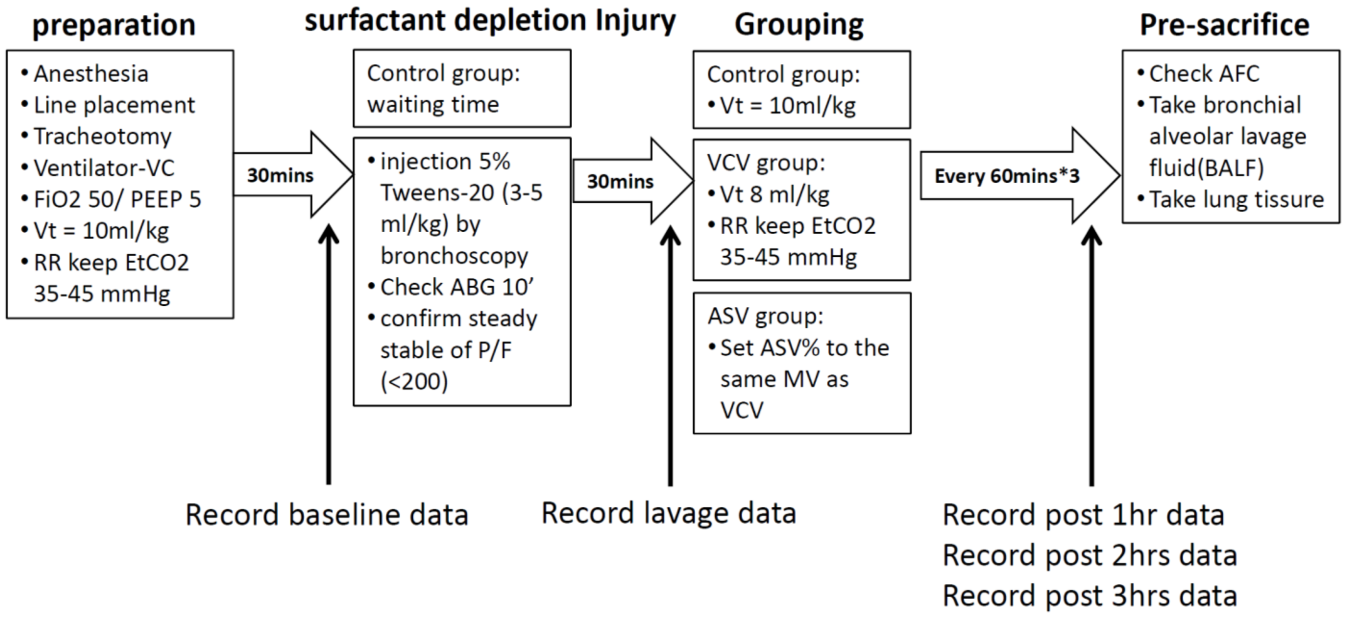

Figure S3. Flow chart showing the experimental design for the animal studies. Before lung injury, prelavage (Baseline) hemodynamics, arterial blood gases and ventilation parameter were measured and recorded. Acute lung injury was induced in VCV and ASV groups. All said parameters were measured and recorded when a stable injury had been achieved (Lavage), subsequently, at 1-hour intervals during the experiment (Post 1hr, Post 2hrs, and Post 3hrs).

Electrical impedance tomography (EIT) measurement:

At each time point, the EIT data(PulmoVista 500, Dräger Medical, Lübeck, Germany) sequence of 1–2 min was recorded and analyzed using special software(EITdiag, Dräger Medical). An EIT electrode belt with 16 electrodes was placed around the thorax at 5 cm above the xiphoid level, and one reference electrocardiogram electrode was placed at the abdomen. The frequency of the injected alternating current was selected automatically according to the noise spectrum. The images were recorded continuously and reconstructed at 40 Hz. The baseline frames for reconstruction were selected as the minimum of the respective EIT data [1].

RESULTS

Human studies

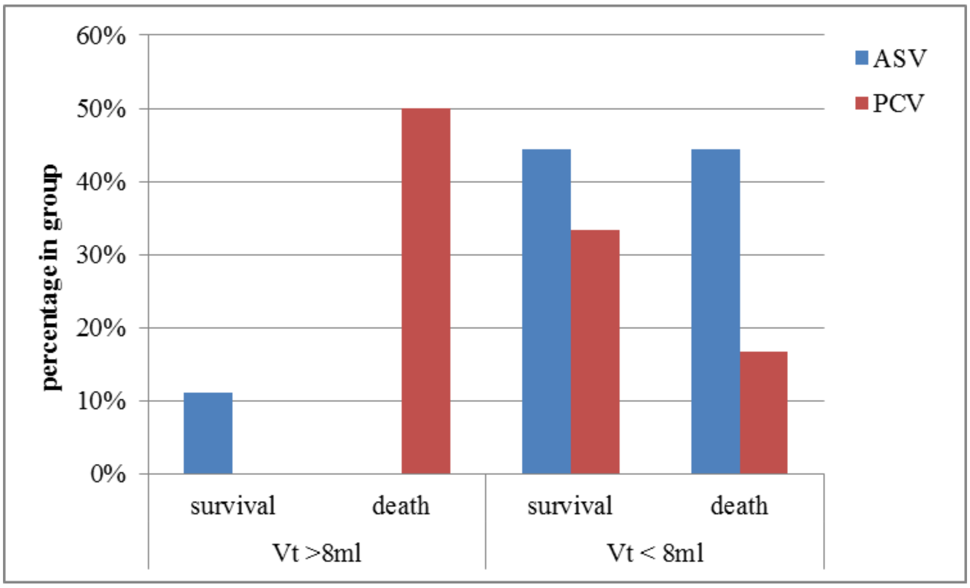

Figure S4. Distribution of Vt <8ml / kg in both groups after 24 hours.

In PCV group, there were 3 cases(50%) > 8 ml//kg, all died; the other 3 cases were <8ml/kg, one died(17%) and two survived(33%). In ASV group, there were 1 case (11%)>8ml/kg is alive and the other 8 cases were <8ml/kg, four died(44.5%) and four survived(44.5%).

Animal experiments

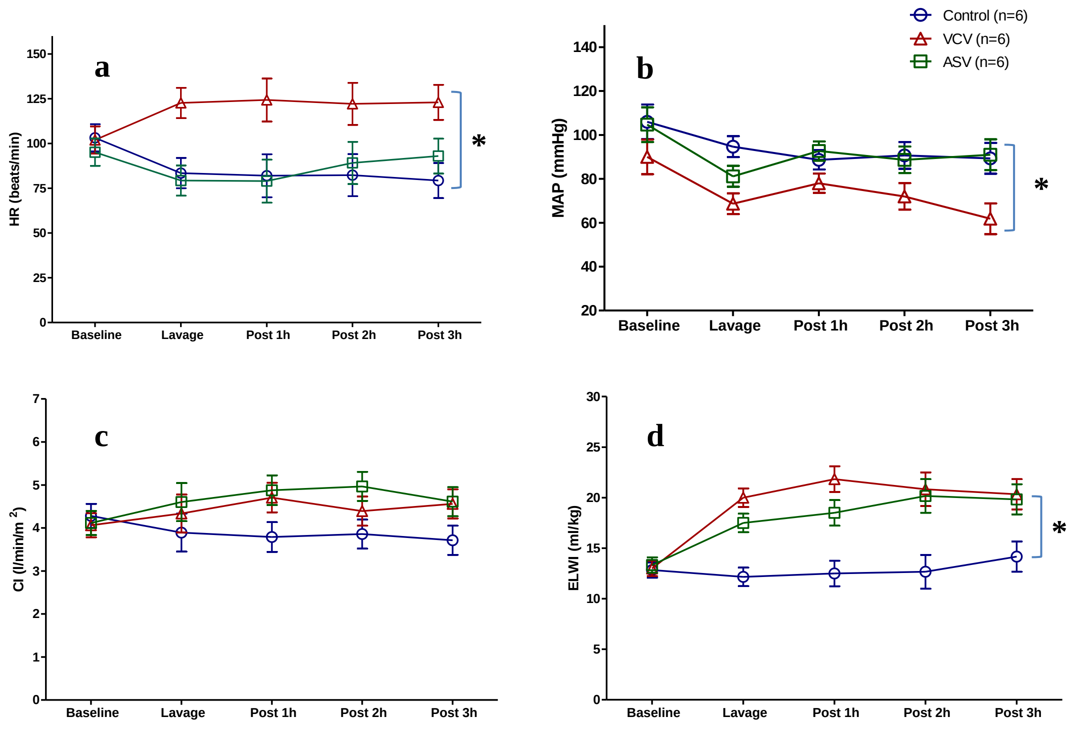

Figure S5. Time course of hemodynamic variables in the three experimental groups (*n* = 6 in each group). (a) heart rate(HR), (b) the mean arterial blood pressure (MAP), (c) cardiac index (CI), (d) extravascular lung water index (ELWI). The data are presented as the mean ± SD. $^{*}p < 0.05$ compared with VCV group.

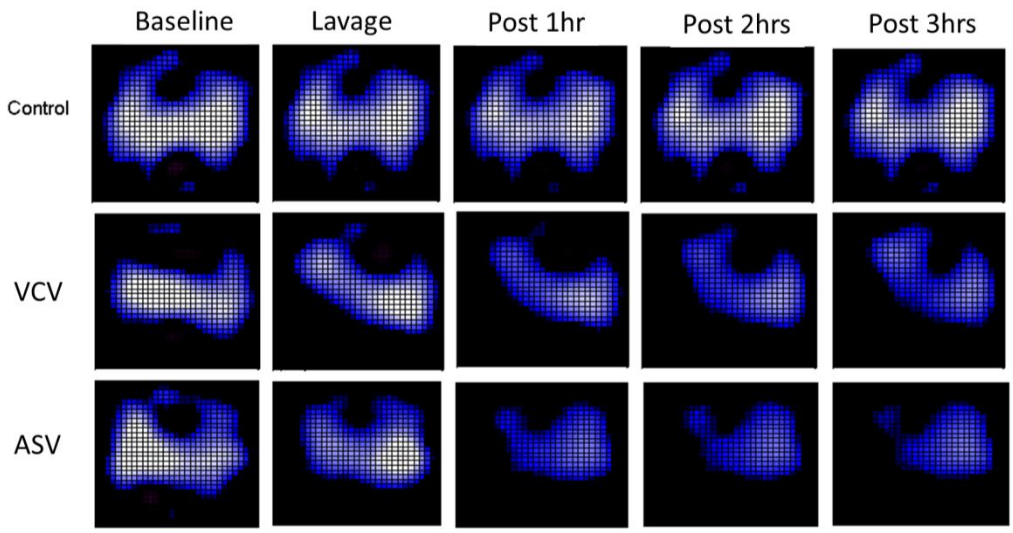

Figure S6. Electrical impedance tomography images in the three experimental groups. (*n* = 1 in each group) Each pixel of the EIT image is blue-tone-encoded. Dark blue pixels describe areas with no ventilation, and white pixels indicate areas with high ventilation.

Table S1. Effects of different time point electrical impedance tomography Data.

Groups	Baseline	Lavage	post 1hr	post 2hrs	post 3hrs
**Control Vt ml/kg**	9.97	10.25	10	9.88	10.19
**Vt% nondep/dep**	48/52	48/52	44/56	48/52	49/51
**ΔEELIgl**	Baseline	36.97	-26.34	3.69	-26.75
**VCV Vt ml/kg**	9.91	10.44	8.2	8.3	8.35
**Vt% nondep/dep**	46/54	46/54	26/74	30/70	44/56
**ΔEELIgl**	Baseline	-5.52	53.23	18.46	-2.01
**ASV Vt ml/kg**	9.79	10	5.11	4.79	4.71
**Vt% nondep/dep**	52/48	51/49	65/35	66/34	65/35
**ΔEELIgl**	Baseline	-46.13	6.46	-130.51	-87.81

Any group n=1, V_T, tidal volume; Vt%nondep = end-inspiratory fraction of tidal ventilation reaching nondependent lung region; Vt%dep = end-inspiratory fraction of tidal ventilation reaching dependent lung region; ΔEELIgl = relative changes of global end-expiratory lung impedance when lavage or post 1-3 hours was considered baseline.

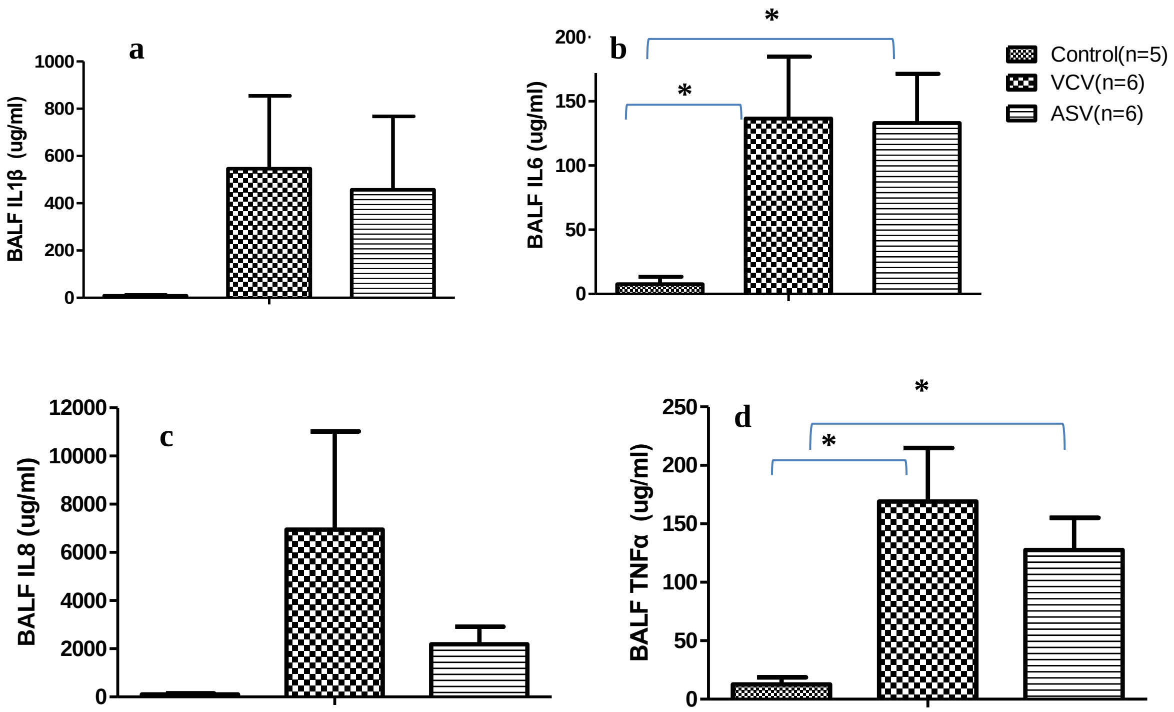

Figure S7. Changes in pro-inflammation cytokines in BALF in animals of control (N=5), VSV (N=6), and ASV (N=6) groups. (A) No statistically significant difference in IL-1β is observed between groups. (B) Statistically significant ($p<0.05$) increase in IL-6 is observed in VCV and ASV groups relative to control group. (C) No statistically significant difference in IL-8 is observed between groups. (D) Statistically significant ($p<0.05$) increase in TNF-α is observed in VCV and ASV groups relative to control group. Data are presented as the average ± the standard deviation.

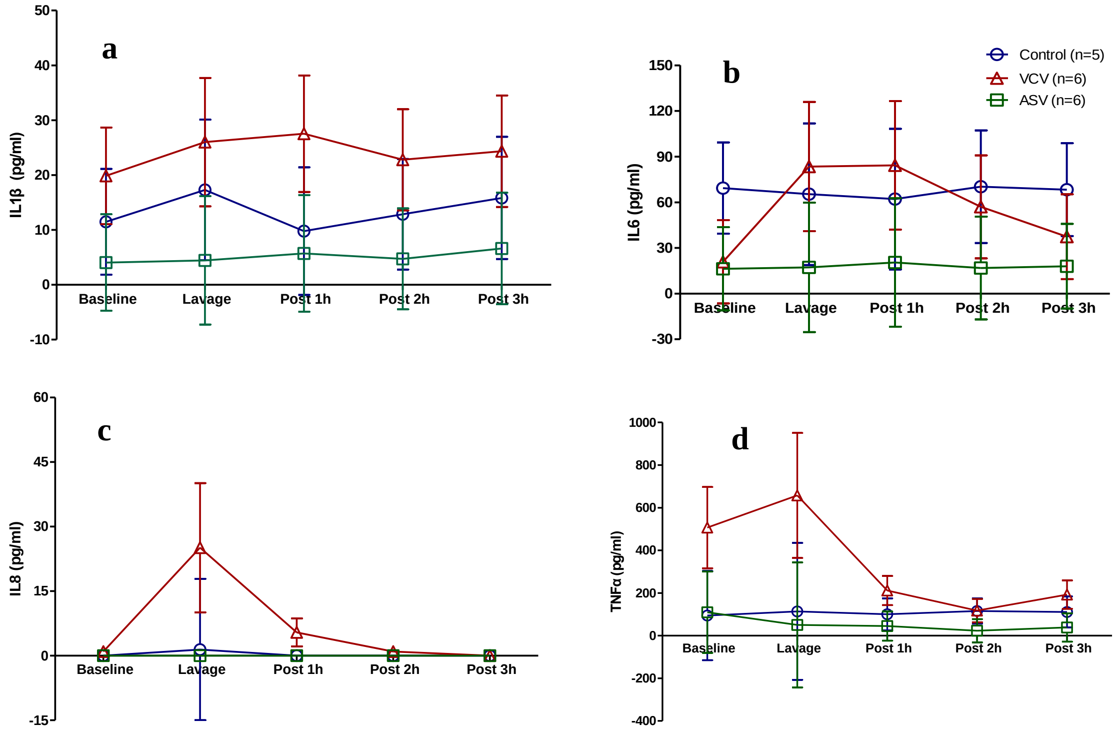

Figure S8. Time course of serum cytokine changes in the three experimental groups, the control groups (N=5), VCV and ASV groups (N=6) there are no statistical differences.

References

1. Liu S, Tan L, Möller K, Frerichs I, Yu T, Liu L, et al. Identification of regional overdistension, recruitment and cyclic alveolar collapse with electrical impedance tomography in an experimental ARDS model. Critical Care 2016;20(1):119.
